# 

**Published:** 1893-01-14

**Authors:** 


**Notices of Births, Deaths, and Marriages,** are inserted in this column at a charge of 2s. 6d. for an announcement not exceeding four lines.

### NOTICE TO CORRESPONDENTS.

All MS., Letters, Books for Review, and other matters intended for the Editor should be addressed THE EDITOR, THE LODGE, PORCHESTER SQUARE, LONDON, W.

The Editor cannot undertake to return rejected M.S., even when accompanied by stamped directed envelope.

**Notice to Advertisers and Subscribers.**

All Advertisements, Orders for Copies of THE HOSPITAL, and Business Communications should be addressed to The Publisher, *not to the Editor*, THE HOSPITAL, 140, Strand, W.C.

The Cost of Subscription, post free, is as follows:—Per week, 2½d.; per quarter, 2s. 6d.; per half-year, 5s.; per annum, 8s. 6d. Foreign:—Per annum, 10s. 8d.; payable, in all cases, in advance.

**"Burdett's Hospital Annual," 1893.**

Fourth year of publication. 700 pp. Boards, gilt lettering. A most complete guide to British, Colonial, Indian, and American Hospitals, Dispensaries, Nursing and Convalescent Institutions, and Asylums. Price 5s. Published by THE SCIENTIFIC PRESS (Limited), 140, Strand, W.C.

**NOTICE.—The Index for Vol. XII. (ending September 1892) is on sale at the Office of this Journal, price 2½d., post free.**

### SCRAPS AND GLEANINGS.

Princess Mary Adelaide, Duchess of Teck has become a patroness of the Central London Ophthalmic Hospital, Gray's Inn Road.

It is intended to hold a festival dinner early in next spring at the Great Northern Hospital, in aid of the funds for the completion of the new buildings.

The total number of patients attending the Devon and Cornwall Throat and Ear Hospital during the past twelve months was 487 against 416 for the preceding year.

The Hospital Saturday collections at Lincoln, Gainsborough, and Sleaford, resulted in the sum of £700 being handed over to the Governors of the Lincoln Hospital.

The Duke of York has given £26 5s. towards the Rebuilding Fund of the Royal Eye Hospital. The total amount received at the opening ceremonial on the 15th inst. was £760.

The Dispensary at Truro lately celebrated its jubilee. Since it was first started, on October 24th, 1842, 30,741 cases have been treated, including 652 admitted during last year.

Of the 5,528 patients treated during the past year at the Coventry and Warwickshire Hospital, no less than 1,200 were accident cases. The in-patients increased by 88, and the out-patients by 1,324.

The Bournemouth Hospital Saturday and Sunday Fund Committee have voted £15 to provide one of Carter's Viaduct Ambulances for the town, and an anonymous donor has given £60 to provide four more.

His Royal Highness the Duke of York has consented to preside at a dinner on behalf of the National Society for the Prevention of Cruelty to Children, to be held at the Hotel Metropole on February 6th.

The Baron de Hirsch has sent to the North West London Hospital, Kentish Town Road, a further donation of £700, making a total of £3,000 he has contributed to this useful and deserving charity since April, 1891.

The Guest Hospital, Dudley, has passed its majority, having been established over twenty-one years. During this period 12,561 patients have been received into the institution. The total number admitted last year was 759.

The London Medical Mission, which provides help and comfort to the poor of St. Giles' in times of sickness and convalescence, is asking for contributions towards its Invalid Kitchen Fund, and also for old and new clothing for its many patients.

The new and splendid building of the Vienna "Poliklinik," a medical college hospital, was recently opened by Archduke Regnier. The institution has been much frequented during the twenty years of its existence by English and American doctors.

Mr. Pinder Simpson, executor of Mrs. E. Hohn, has forwarded a cheque for £100 to the Hospital for Epilepsy, Regent's Park. The hospital has also recently received handsome presents of game from the Prince of Wales and the Duke of Northumberland.

The Mayor of Birmingham, presiding at the annual meeting of the Dental Hospital, said that the operations had increased something like 10 per cent., being 19,274 for the past year as against 17,056 in the previous year. The income was £581 in 1891, and only £526 in 1892.

The Committee of the Salford Football Club have promised to subscribe £50 yearly for the support of one of the beds in the Salford Royal Hospital. A cot in the children's ward has been named the "Anne Sykes Cot," in memory of Mrs. Sykes, who left a legacy of £1,000 to the institution.

The abolition of the recommended system has been agreed upon by the governors of the Salford Royal Hospital, and during the present year they have decided to try the experiment of admitting to the benefits of the charity all persons too poor to be able to afford to pay for medical attendance and medicine, and who are not in receipt of Poor Law relief.

The Ham Yard Soup Kitchen and Hospice supplied, from October 1st, 1891, to September 30th, 1892, meals to 148,280 families on the Register, and gave away 145 tons 12½ cwt. of coals during the winter months. The number of penny dinners provided during the year was 73,748, and the receipts £307 5s. 8d. On Christmas Day 1,019 families were supplied with a good Christmas dinner.

At Whitsuntide, the trustees of the Bellahousten Fund, a sum of about £500,000, bequeathed to the City of Glasgow by the late Misses Steven, of Bellahousten, have determined to distribute £64,250 among 37 charities. The three infirmaries, the Royal, the Western, and the Victoria are to receive £10,000 each. The trustees have also resolved to distribute annually a sum not exceeding £1,000 among various minor charities.

At the Belfast Royal Hospital 2,322 new cases were received into the wards for treatment during the year ending August 31st, 1892, whilst 133 cases which remained in the hospital from the previous year made a total of 2,455, the largest number of in-patients ever treated in one year at the hospital. The extern cases amounted to 20,293. The year was started with a debt of £2,283 15s. 6d., but this has been cleared off, and the receipts have considerably exceeded the debt and the expenditure.

The East London Hospital for Children contains, apart from the accident ward and dispensary, 102 cots in its boys, girls, and infants' wards. The great feature of the past half-year's work is the completion and use of the new out-patient rooms. The committee feel the great need of establishing a convalescent home in connection with the hospital. Towards this object £1,000 has been promised by Mr. Galpin, an old friend of the hospital, and £800 by other friends. Altogether the sum of £3,000 is required.

The Burial Reform Association have resolved to form a Church Sanitary Association, whose aim should be to aid in ensuring to everyone pure air, pure water, a wholesome dwelling, and surroundings safe-guarded from preventable disease, with the Church House as its chief office. The following gentlemen have promised to read papers on successive first Thursdays in the month, at the Church House: Sir Spencer Wells, Bart., the Archdeacon of London, Mr. Ernest Hart, Editor of the *British Medical Journal*, the Rev. J. F. Kitto, Dr. G. V. Poore, and Mr. Fred. Scott.

### THE STUDENT OF MEDICINE.

[All communications for this Supplement should be addressed to THE EDITOR of THE HOSPITAL, at 140, Strand, with the words, "Students' Column," written in the left hand top corner of the envelope.]

### APPOINTMENTS.

Perry, Edwin Cooper, B.A.Camb., M.D., M.R.C.P., M.R.C.S., appointed Medical Superintendent to Guy's Hospital. Richardson, Richard T., M.R.C.S.Eng., appointed Medical Officer of Health for Trowbridge. Riley, James Woodward, M.R.C.S. Eng., L.R.C.P.Edin., appointed Medical Officer and Public Vaccinator to the Pontesbury District of the Atcham Union. Sinclair, F. H., M.D., M.Ch., L.R.C.P., L.R.C.S.I., re-appointed Honorary Attending Physician to the Belfast Hospital for Consumption and Diseases of the Chest. Stack, E. C., L.R.C.P., L.M., L.R.C.S.I., appointed Honorary Attending Surgeon to the Belfast Hospital for Consumption and Diseases of the Chest. Symmers, William St. Clair, M.D.Aberd., appointed Pathologist to the General Hospital, Birmingham. Thomas, John T., L.R.C.P.I., L.R.C.S.Edin., appointed Medical Officer for the Magor District of the Newport (Mon.) Union. Vardon, A. D., L.R.C.P., L.R.C.S.Edin., L.F.P.S.Glas., appointed Medical Officer for the Eastern Sanitary District of the Gamrie Parochial Board. Warrington, W. B., M.B., B.Ch., Vict. Univ., appointed House Physician to the City of London Hospital for Diseases of the Chest, Victoria Park. Woollett, Sidney Winslow, M.R.C.S.Eng., L.S.A., appointed Medical Officer for the Wangford District of the Blything Union. Barron, Barclay Josiah, M.B., C.M.Edin., appointed Physician to the Nose and Throat Department of the Bristol General Hospital. Biernacki, John, M.B., C.M.Glas., appointed Medical Officer of the Bootle Borough Hospital. Bonar, Thomas Mitchell, M.B., C.M.Glas., appointed Medical Officer of Health for the Western District of the Truro Rural Sanitary Authority. Boyd, John St. Clair, M.B., M.Ch., R.U.I., appointed Surgeon to the Samaritan Hospital for Women, Belfast. Brown, John, M.D.Vict., M.B., Ch.B., re-appointed Medical Officer of Health for Bacup. Bury, R. F., appointed House Surgeon to St. George's Hospital. Clarke, J. Michell, B.A.Camb., M.D., M.R.C.S., appointed Physician to the Bristol General Hospital. Crosse, Herbert William, M.B., C.M.Edin., appointed temporary Medical Officer of Health for Norwich. Elliott, C. N., M.B., C.M.Dub., appointed Medical Officer of Health for the Rural Sanitary District of the Oundle Union. Ellis, W. C., L.R.C.P., L.R.C.S.Edin., L.F.P.S.Glas., appointed Medical Officer for the Tollerton Sanitary District of the Great Ouseburn Union. Fullarton, Robert, M.D., appointed Lecturer on Diseases of the Throat and Nose at St. Mungo's College, Glasgow. Goodwin, Edward Knot, M.R.C.S., L.R.C.P.Lond., appointed Assistant House Surgeon to the Royal Albert Hospital and Eye Infirmary, Devonport. Harrison, Reginald, F.R.C.S., appointed House Surgeon to the Kingston Provident Dispensary. Heaven, John C., L.R.C.P., M.R.C.S., L.S.A., D.P.H., re-appointed Medical Officer of Health to the Keynsham Rural Sanitary District. Kent, Robert Thomas, M.A., F.R.C.S., appointed Professor of Anatomy at St. Mungo's College, Glasgow. Lancaster, Ernest Le Cronier, B.A.Oxon., M.B., B.Ch., appointed Medical Officer to the Outdoor Patients at the Swansea Hospital. Little, Andrew, M.B., C.M.Aberd., appointed Ophthalmic and Aural Surgeon to the Victoria Hospital, Burnley. Lush, Percy J. F., M.A., M.B., B.Ch.Oxon., M.R.C.S., appointed Medical Officer of Friedenheim, Upper Avenue Road, Swiss Cottage, N.W. Maclure, Herbert William, B.A.Camb., M.B., B.C., appointed Medical Officer for the South District of the Paddington Union. Marriott, Osburne Delano, M.D.Glas., re-appointed Medical Officer for Seventh District of the Sevenoaks Union. Overend, Mr. W., appointed Junior Assistant Medical Officer to the Infirmary of the Fulham Union. Parker, Geo., M.A., M.D.Cantab., M.R.C.S.Eng., appointed Assistant Physician to the Bristol General Hospital. Robertson, George M., M.B., C.M.Edin., appointed Medical Superintendent of the Perth District Asylum at Murthly. Romer, R. Leslie, appointed Honorary Physician to St. George's Hospital. Sibley, W. Knowsley, M.A., M.D., B.C., M.R.C.P., appointed Clinical Assistant to the Skin Department of the Middlesex Hospital. Snell, Mr. E. H., appointed Assistant Medical Officer at the Workhouse and Infirmary of the Parish of Paddington. Walker, Benjamin, M.D.Durh., M.R.C.S.Eng., re-appointed Medical Officer for the Ravenstonedale District of the East Ward Union. Williams, W., M.A., M.B., B.S.,

### The Hospital Advertiser.

### MEDICAL APPOINTMENTS.

### NORTH STAFFORDSHIRE INFIRMARY AND EYE HOSPITAL.

Salary £80 per annum, with Board, Lodging, and Washing.

Candidates, who must be duly qualified and registered, must forward their applications to the SECRETARY, stating their qualifications and enclosing testimonials, on or before 24th January, 1893.

The House Surgeon will be required to lecture to the nursing probationers.

The successful candidate must be prepared to enter on his duties on the 1st March, 1893.

EUGENE E. STREET, Secretary.

The Infirmary, Chichester,

Dec. 13th, 1892. (29)

### VICTORIA HOSPITAL, FOLKESTONE.

Norfolk House, Norfolk Street, Strand, W.C.,

24th December, 1892. (1,621)

### DERBYSHIRE ROYAL INFIRMARY.

THE STUDENT OF MEDICINE—(*continued*).

D.P.H.Oxon., M.R.C.S.Eng., F.C.S.Lond., appointed Medical Officer of Health to the Glamorganshire County Council. Wilson, John, L.R.C.P., L.R.C.S.Edin., re-appointed Medical Officer of Health for Pudsey. Winstanley, R. W., L.R.C.P., L.M.Edin., M.R.C.S., appointed Medical Officer for the Shotter Mill Sanitary District of the Farnham Union. Mr. Archibald Dukes, St. Thomas's Hospital, has been appointed House Surgeon to the Croydon General Hospital.

### VACANCIES.

The following vacancies are announced this week, the amount of salary in each case being given after the name of the institution:

### Resident Medical Officers.

Alnwick Infirmary. House Surgeon. Unmarried. Salary £120 per annum, with furnished apartments, attendance, coal, and gas. Applications to W. T. Hindmarsh, 26, Bondgate Without, Alnwick, by January 14th.

Chichester Infirmary, Chichester. House Surgeon. Salary £80 per annum, with board, lodging, and washing. Applications to E. E. Street, Secretary, by March 1st.

Derbyshire Royal Infirmary. Assistant House Surgeon. Appointment for six months, but eligible for re-election for additional six months. Salary, £10 for first six months, £25 for second six months, with separate apartments, board, and washing. Applications to the Secretary by January 19th.

East London Hospital for Sick Children. House Surgeon. Board and Lodging, but no salary. Must be registered. Applications to Secretary by January 25th.

Killarney District Lunatic Asylum. Assistant Medical Officer. Salary £100 per annum, with allowances valued at £100 yearly. Candidates must be unmarried, and not over 30. Applications to Dr. L. T. Griffin, Resident Medical Superintendent. Election on January 19th.

Metropolitan Asylums Board, Norfolk House, Norfolk Street, Strand, W.C. Medical Superintendent at the Darenth School for Imbeciles, near Dartford, Kent. Doubly qualified. Not more than 45 years of age. Salary, £450 per annum, with furnished residence, coals, gas, milk, garden produce, and washing. Applications, on forms to be obtained at the Office of the Board, to be sent in by January 23rd.

Miller Hospital and Royal Kent Dispensary, Greenwich, S.E. Junior Resident Medical Officer. Salary, £30 per annum, with board, attendance, and washing. Applications to the Honorary Secretary by January 14th.

North Staffordshire Infirmary and Eye Hospital, Hartshill, Stoke-on-Trent. House Surgeon; doubly qualified. Salary £120 per annum, increasing £10 yearly, with furnished apartments, board, and washing. Applications to the Secretary by January 21st.

Taunton and Somerset Hospital, Taunton. Assistant House-Surgeon; appointment for six months. Board, washing, and lodging provided. Applications, endorsed "Assistant House-Surgeon," to the House-Surgeon by January 18th.

Victoria Hospital Folkestone. House-Surgeon, unmarried. Salary £80 the first year, with an increase of £10 each year for the two following years. Applications to the Secretary by January 21st.

Victoria Hospital for Sick Children, Queen's Road, Chelsea, S.W. House Physician and House Surgeon to the In-patients; doubly qualified. Honorarium £50 per annum, with board and lodging. Applications to the Secretary by January 14th.

West Herts Infirmary, Hemel Hempstead. House Surgeon and Dispenser, doubly qualified, unmarried. Salary £100 per annum, with board, furnished rooms, light, fire, attendance, and washing. Applications to the Secretary by January 31st.

### Non-Resident Medical Officers.

Corporation of Norwich. Medical Officer of Health. Salary, £400 per annum. Applications, endorsed "Medical Officer of Health," to Geo. B. Kennett, Town Clerk, Guildhall, Norwich, by January 28th.

St. George's Hospital, S.W. Visiting Apothecary. Applications to the Secretary by January 18th.

Stratford-on-Avon Rural Sanitary Authority. Medical Officer of Health. Salary £110 per annum. Applications to John Charles Warden, 9, Guild Street, Stratford-on-Avon, by January 21st.

Tullaroan Dispensary, Kilkenny. Medical Officer for the Tullaroan Dispensary District. Salary £100 per annum, and £20 as Medical Officer of Health, with usual vaccination and registration fees. Applications to Mr. Edmond Walsh, Honorary Secretary, Committee Room, Tullaroan, before January 16th.

University College of South Wales and Monmouthshire, Cardiff. A Professor of Anatomy, and a Professor of Physiology. Stipend in each case £350 per annum. Applications to Ivor James, Registrar, by February 10th.

University of Edinburgh. Examiner in each of the following subjects: (1) Anatomy, (2) Chemistry and Laboratory Work for the First B.Sc. Examination in Public Health, (3) Midwifery, (4) Practice of Physic, and (5) Botany. The salary in each of the departments Nos. 1, 3, 4, and 5 is £75 a year, and in No. 2 £90 a year, with an allowance of £10 per annum for travelling and other expenses in the case of Examiners not resident in Edinburgh or the immediate neighbourhood. Applications to M. C. Taylor, Secretary.

University of Glasgow. Assistant Examiner in Zoology. Salary £30 per annum. Applications to the Secretary of the Court, Mr. Alan E. Clapperton, West Regent Street, Glasgow.

The Hospital Advertiser.

MEDICAL APPOINTMENTS—(*Continued*).

EAST LONDON HOSPITAL FOR CHILDREN,

Glamis Road, Shadwell, E.

HOUSE SURGEON.

